# Population Genomics Approaches Identify a Cryptic, Emerging Generalist Pest Complex

**DOI:** 10.1002/ece3.74347

**Published:** 2026-09-11

**Authors:** Pedro A. P. Rodrigues, Michael S. Crossley, Benjamin L. Aigner, William Rodney Cooper, David Scott Carlson, Pamela Sapp, Mark R. Abney, William E. Snyder

**Affiliations:** ^1^ Department of Entomology University of Georgia Athens Georgia USA; ^2^ Department of Entomology and Wildlife Ecology University of Delaware Newark Delaware USA; ^3^ Department of Entomology University of Georgia Tifton Georgia USA; ^4^ Temperate Tree Fruit and Vegetable Research Unit USDA‐Agricultural Research Service Wapato Washington USA; ^5^ Worth County Extension University of Georgia Sylvester Georgia USA; ^6^ Jefferson County Extension University of Georgia Louisville Georgia USA

**Keywords:** burrower bug, climate change, cryptic species, molecular gut content analysis, peanut, rapid response

## Abstract

Information about biological traits essential for pest management, such as species identity, diet and movement often require laborious and time‐intensive studies on pest natural history, in both laboratory and field settings. However, new agricultural pest threats are continually emerging, often requiring prompt responses with limited information. Using a combination of molecular gut content analysis and RAD‐seq, we examined the species identities, plant diet composition, and population genetic structure of an emerging and important agricultural pest in the US, the peanut burrower bug, *Pangaeus bilineatus* Say (Hemiptera, Cydnidae). We found that two, morphologically similar, burrowing bug species (including *P. bilineatus* ) were commonly caught in light traps near peanut fields, one of which ( *Dallasiellus lugubris* ) was not previously considered a pest of peanut. Molecular gut content analysis revealed a wide, but somewhat distinct, variety of plants among the diets of both bug species. Surprisingly, peanut was a rare part of the diet of either species. RAD‐seq analysis revealed evidence consistent with weak isolation‐by‐distance and modest spatial genetic differentiation for both species. Together, these results suggest a potential pest complex where previously only one species was in focus. Moreover, their broad diets and spatially restricted population dispersal patterns may also explain the sporadic nature of damage that has been recorded for this potential pest complex. Responses to emerging pest challenges can benefit from insights generated by population genomics techniques, opening up new avenues for research and supporting efforts to quickly tailor management strategies for novel pests.

## Graphical abstract

Molecular gut content analysis and RAD‐seq analysis revealed a peanut pest complex where previously only one species was considered problematic. Both species exhibited a wide, but somewhat distinct, variety of plants among their diets, and peanut‐feeding was rare. Diet generalism combined with a stepwise pattern of dispersal among fields might underlie the sporadic nature of peanut damage by this pest complex.

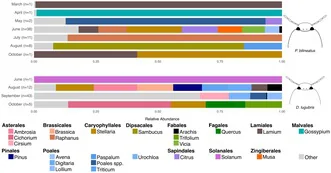

## 1 Introduction

New agricultural insect pest threats are continually emerging (Paini et al. 2016; Lehmann et al. 2020), and knowledge about their biology and ecology is needed, often in short order, to be able to respond effectively. Some of the most common methods for studying pest insects' biology, however, can be time‐ and/or cost‐intensive. For instance, to determine host suitability and diet breadth, extensive field observations and greenhouse experiments are often needed (Muñiz 2000; Fordyce et al. 2016). To determine insect dispersal, mark‐and‐recapture and field observations depend on choice of marking dye/technique, weather events, multiple visits to field sites, and knowledge on insect peak activity (Hagler and Jackson 2001; Weldon et al. 2014). Approaches such as molecular gut content analysis and reduced‐representation genome sequencing (e.g., RAD‐seq), on the other hand, could save time and be more cost‐effective alternatives for quickly assessing diet breadth and population structure of emerging threats.

Molecular gut content analysis utilizes observations of species' DNA in the digestive tract of organisms to infer diet composition, often with highly efficient next‐generation sequencing technology and bioinformatics pipelines (Pompanon et al. 2012). Molecular gut content analysis methods are powerful tools for uncovering interactions in pest species trophic webs. For instance, Lynch et al. (2022) found that DNA from the Colorado potato beetle *Leptinotarsa decemlineata* is more often detected in the generalist predator *Nabis* sp. collected in conventional farms compared to organic farms, whereas detection is higher in organic rather than conventional farms for co‐occurring predators in the genus *Geocoris* sp. Plant DNA is also detectable in herbivorous insects: sequencing of plant DNA from phloem‐feeders such as *Bactericera cockerelli* revealed a greater range of previously unknown host species that was further validated by greenhouse experiments (Cooper et al. 2023).

Other molecular tools, such as RAD‐seq, can help infer population and metapopulation structure and movement over time (Fu et al. 2017). RAD‐seq allows the identification of DNA sequence variants by adopting a reduced‐representation sequencing of the genome, i.e., inferences are made based on shearing and filtering the genome by a target size, such that only a subset of the genome is sequenced. In cotton systems, for instance, analysis of RAD‐seq data revealed gene flow among populations of the fleahopper *Pseudatomoscelis seriatus* collected in cotton, wooly croton, and other wild plant hosts, supporting previous observations that indicated movement among these hosts within and between seasons (Raszick et al. 2020). Moreover, RAD‐seq data may also help identify species complexes (Doorenweerd et al. 2024), which may be missed by agents without species‐specific taxonomical training.

The peanut burrower bug ( *Pangaeus bilineatus* Say; Hemiptera: Cydnidae) recently emerged as an important pest of peanut ( *Arachis hypogaea* L.) in the Southeastern USA, but the sporadic nature of damage presents a challenge to management. Here, we sought to assess the degree of host plant specificity and dispersal through the landscape of peanut burrower bugs in the peanut‐producing region of south Georgia, USA. We did so using a combination of molecular gut content analysis and RAD‐seq to examine the species identities, plant diet composition, and population genetic structure of *P. bilineatus* . In addition, we also sampled another species, *Dallasiellus lugubris* Stål (Hemiptera: Cydnidae). The biology of this species is also poorly known, and it is unknown whether it uses peanuts as its host plant. We characterized its identity, host range, and population structure as well, to find out whether *D. lugubris* and *P. bilineatus* also share similarities in their diet breadth and population structure.

## 2 Materials and Methods

### 2.1 Study System

*Pangaeus bilineatus* is native to Central and North America, being described from collections in the eastern US as early as 1824 (Froeschner 1960). Although peanut cultivation was widespread in the US by 1890 (Crossley et al. 2021), *P. bilineatus* was not reported as a peanut pest until light infestations were noticed in Georgia in 1959 (Smith and Pitts 1974). Severe economic losses occurred during a 1966 outbreak in Alabama peanuts, and economic losses have been reported in other southern US states since (Aigner et al. 2021). While being considered only a sporadic pest throughout peanut producing areas, since 2010 it has caused significant losses in Georgia, USA, where half of US peanuts are produced (Aigner et al. 2021). When burrower bug‐caused injury to peanut exceeds 3.49%, the entire load of peanuts is downgraded from “segregation I” to “segregation II”, representing a substantial loss of revenue (Aigner et al. 2021). The reason for *P. bilineatus* ' sudden elevation to pest status is unknown, but could be related to more widespread planting of preferred/susceptible peanut cultivars, increasing adoption of reduced tillage practices, or more frequent periods of drought interacting with insect feeding to increase damage on cultivated crops (Kennedy 2011; Aigner et al. 2021).

While *P. bilineatus* is by far the most abundant burrowing bug species noted in commercial peanut fields, it can co‐occur with other species, including *Pangaeus congruous* (Uhler), *Cyrtomenus ciliatus* (Palisot de Beauvois), *Sehirus cinctus* (Palisot de Beauvois), and *Tominotus communis* (Uhler) (Smith and Pitts 1974; Chapin and Thomas 2003; Schwertner and Nardi 2015). Given their dominance in peanut fields, economic losses in peanut are usually attributed to feeding by *P. bilineatus* , with just one report including *T. communis* as a culprit in a damaging burrowing bug outbreak in Alabama (Smith and Pitts 1974). However, *P. bilineatus* is by no means a specialist on peanut. It has been documented feeding on seeds, stems, or roots of a variety of crops in addition to peanut, including cotton (*Gossipium hirsutum* L.), pepper (*Capsicum* spp. L.), spinach ( *Spinacia oleracea* L.), strawberry (*Fragaraia ananassa* Duchesne), and wheat ( *Triticum aestivum* L.). It also utilizes a variety of non‐crop hosts including chickweed ( *Stellaria media* (L.) Villars), river oats ( *Chasmanthium latifolium* (Michx.) Yates), white clover ( *Trifolium repens* L.), and yellow nutsedge ( *Cyperus esculentus* L.) (Aigner et al. 2021). Still, the frequency of these plants in the diet of *P. bilineatus* is unknown.

### 2.2 Specimens and DNA Extraction

Light traps were installed near peanut fields in Randolph County and Worth County (Georgia, USA) and checked at weekly intervals. In 2020, two traps were placed in one site in Randolph County (one at the center pivot and one along the edge of the field), and individual traps were placed at the edge of two fields in Worth County. In 2021, individual traps were placed at the edge of two fields in Worth County. To provide a broader geographic sample for assessment of population structure, additional hand‐collected samples were included from individual fields in Emanuel County, Jefferson County, and Worth County in 2019. Light traps (BioQuip model 2851A‐M) were suspended from wooded posts placed in and adjacent to peanut fields such that the bottom of the trap was approximately level with the top of the peanut canopy. A single, 12 V, circular fluorescent light bulb powered by a rechargeable battery/solar panel system and controlled by a digital timer was set to turn on daily at 20:00 h and turn off at 0:00 h. Samples were recovered weekly, and at each inspection, samples were collected in 70% ethanol and stored in a −20°C freezer until specimens were sorted and burrower bugs identified with the aid of a stereo‐microscope (gross morphology) and molecular methods (see “3. Molecular validation of species identification,” below). Light traps were operated between April and October (peanut growing season), and burrower bugs were encountered in light traps during the months of April, May, June, August, September, and October in 2020, and July, August, September, and October 2021 (a map depicting sample sites is available in Figure 4). A total of 127 *P. bilineatus* and 123 *D. lugubris* individuals were obtained for subsequent RAD‐seq and/or molecular gut content analysis. Prior to DNA extraction using DNEasy Blood and Tissue kit (Qiagen, Germany), whole insects were rinsed with 100% ethanol then crushed and homogenized with a sterile micropestle in 180 uL ATL buffer. Samples were then processed according to the manufacturer's protocol, with the following modifications: 4 μL of RNase A (Qiagen, Germany) was added during overnight incubation with proteinase K, and the final elution step was done using 80 μL AE buffer. Samples were then stored in a −20°C freezer until sequencing. DNA quality was assessed using a NanoDrop spectrophotometer. In addition, to validate the ability to detect peanut DNA in burrower bugs, *P. bilineatus* were also reared for 2–3 generations on peanuts in salad containers with perforated lids and filled halfway with field‐collected sand, kept at ~24°C and ambient light and humidity.

### 2.3 RAD‐Seq Sequencing and Population Structure Analysis

Genomic DNA was converted into nextRAD genotyping‐by‐sequencing libraries (SNPsaurus LLC) (Russello et al. 2015). Genomic DNA was first fragmented with Nextera Flex reagent (Illumina Inc), which also ligates short adapter sequences to the ends of the fragments. The Nextera reaction was scaled for fragmenting 24 ng of genomic DNA, although 36 ng of genomic DNA was used for input to compensate for the amount of degraded DNA in the samples and to increase fragment sizes. Fragmented DNA was then amplified for 27 cycles at 74°C, with one of the primers matching the adapter and extending 10 nucleotides into the genomic DNA with the selective sequence GTGTAGAGCC. Thus, only fragments starting with a sequence that can be hybridized by the selective sequence of the primer will be efficiently amplified. The nextRAD libraries were sequenced on a Novaseq 6000 with one SP lane of 122 bp single‐end reads (University of Oregon).

We identified single nucleotide polymorphisms (SNPs) from RAD‐seq data for each species using Stacks 2 (Rochette et al. 2019), using the denovo_map.pl. program (which builds loci *de novo*, instead of after aligning to a reference genome), using a minimum minor allele frequency threshold of 0.05 (‐‐min‐maf 0.05), requiring a locus to be present in at least 30% of samples (‐‐min‐samples‐per‐pop 0.3) in all populations (‐‐min‐populations 9 or 6 depending on species), and retaining only one SNP when multiple were detected on a given locus (‐‐write‐random‐snp). Default settings were used for remaining parameters (e.g., ‐m = 3 and ‐M = 2). This resulted in VCF files with 6151 SNPs for *P. bilineatus* , and 9590 SNPs for *D. lugubris* . SNP genotypes in VCF files were imported into R using custom scripts. Insect samples were removed from analysis that had at least 30% missing SNP genotypes, resulting in removal of 15 *P. bilineatus* samples and 18 *D. lugubris* samples. We then removed SNPs that had at least 50% missing genotypes, or that were monomorphic after removing samples with > 30% missing genotypes, resulting in removal of 377 and 745 SNPs from *P. bilineatus* and *D. lugubris* datasets, respectively. Thus, after filtering, we retained 5774 loci and 113 samples of *P. bilineatus* , and 8845 loci and 105 samples of *D. lugubris* . The resulting *P. bilineatus* dataset contained 19.3% ± 0.04% (range 2.5%–28.0%) missing genotypes, while the resulting *D. lugubris* dataset contained 19.3% ± 0.03% (range 15.0%–28.4%) missing genotypes. Depth of coverage was assessed using the vcfR R package (Knaus and Grünwald 2017). The mean depth of coverage across *P. bilineatus* samples was 65.9× (range 5.8×–556.4×), with 79.5% of SNPs having > 20× mean coverage. The mean depth of coverage across *D. lugubris* samples was 78.1× (range 7.4×–542.5×), with 89.4% of SNPs having > 20× mean coverage. We then summarized and visualized genetic differentiation among individual insects using Principal Components Analysis. Because PCA cannot handle missing values, we first imputed missing genotypes using the mode for each SNP locus.

To further examine spatial patterns of genetic variation, we conducted a spatial Principal Components Analysis (sPCA) using the adegenet R package (Jombart et al. 2008; Jombart and Ahmed 2011). The filtered SNP datasets were converted to genind objects, and individual geographic coordinates were used to define spatial relationships among samples. Spatial connections among individuals were established using a Delaunay triangulation network. Because duplicate sampling coordinates prevent construction of Delaunay networks, a small random jitter was applied to coordinates prior to network generation. Spatial principal components were then calculated using the spca function in adegenet. Significance of global spatial genetic structure (positive spatial autocorrelation) and local spatial genetic structure (negative spatial autocorrelation) was evaluated using Monte Carlo permutation tests with 199 permutations implemented in spca_randtest. This function was modified to correctly compute one global and one local test statistic per permutation, as the distributed implementation collapsed all permutation replicates into a single value for the tests. The axis‐specific eigentests remained unchanged. Following Jombart et al. (2008), interpretation focused on significant global and local patterns identified by the permutation tests and the leading positive and negative sPCA axes.

To examine how genetic differentiation among populations varied with distance, we first calculated Weir and Cockerham *F*_ST_ among populations using calculate.pairwise.*F*st in the BEDASSLE R package (Bradburd et al. 2013). A small number of SNPs were found to be monomorphic after imputation (1 for *P. bilineatus* , 18 for *D. lugubris* ) and were removed prior to calculation of *F*_ST_. We calculated pairwise geographic distances (km) among populations using functions available in the terra R package (Hijmans et al. 2026) using R version 4.5.2 (R Core Team 2025) then compared *F*_ST_ and log‐transformed geographic distance using a Mantel test using the vegan R package (Oksanen et al. 2026).

### 2.4 Molecular Validation of Species Identification

Species identification was validated for samples that were also submitted for RAD‐seq sequencing. Before this publication, we were unable to find any genomic information for *D. lugubris* in online databases (e.g., NCBI and BOLD), whereas *COI* sequences already existed for *P. bilineatus* (e.g., NCBI, acc. KR031248.1), which helped validate our *P. bilineatus* draft genome identification. For this reason, RAD‐seq data were aligned against draft genomes that we sequenced for *P. bilineatus* and *D. lugubris* , using Bowtie2 (*k* = 20, Langmead and Salzberg 2012). The *P. bilineatus* draft genome had an assembly size of 1083.0 Mb, 584,742 scaffolds, scaffold N50 of 7.95 Kb, longest scaffold of 366.1 kb, and GC content of 31.4%, determined using BBtools (Bushnell 2014). The *D. lugubris* draft genome had an assembly size of 1136.9 Mb, 615,441 scaffolds, scaffold N50 of 9.08 kb, longest scaffold of 156.7 kb, and GC content of 32.9%. These draft genome assemblies were generated to provide reference sequences for species identification, and were not intended as chromosome‐level genome assemblies. Using Samtools (Danecek et al. 2021) and custom scripts, results from Bowtie2 were summarized for all samples, and a species identification was assigned to samples according to the best alignment rate (greater or equal to 50%) to one of the reference genomes. In cases where alignment was low (< 50%) to either of the reference genomes, these samples were classified as “unknown” and excluded from the dataset.

To further validate our *D. lugubris* draft genome and samples, we barcoded (*COI*) thirty samples collected among our samples collected in 2021: 27 samples identified as *D. lugubris* , 2 samples identified as *P. bilineatus* , and 1 sample identified as “unknown” (i.e., low alignment rate to either of our reference draft genomes). In addition, six additional samples collected in 2022 and identified as *D. lugubris* by Mark Abney (University of Georgia) were also added to generate the first *COI* sequences ever to be added to the NCBI and BOLD databases. A 418 bp long region of the *COI* gene was amplified using the BF3‐BR2 primers, as described in Elbrecht et al. (2019), and submitted for Sanger sequencing (Eurofins Genomics LLC, USA) in both directions. Sequences were quality‐trimmed in Geneious Prime (version 2023.2.1, Build 2023‐07‐20; error probability for quality trimming set to 0.05), primers were identified and removed, and forward and reverse sequences were then assembled. We excluded sequences from our analysis in cases where ambiguity could not be resolved. Our “unknown” species sample was discarded due to low‐quality sequences, and it was replaced by sample 1930‐1 that had already been sequenced, using a different primer set (primers Lep2t1‐LepR1, Park et al. 2010) targeting a *COI* region that overlaps with the region targeted by BF3‐BR2. For building the phylogenetic tree, we added *COI* sequences representing other, related burrower bug taxa (e.g., *Sehirus*, *Cyrtomenus*, and *Tominotus*), close matches found via Blast (NCBI, nt/nr database; “Cydnidae”) and published *Pangaeus bilineatus* sequences. All sequences in the dataset were then aligned against KR031248.1, trimmed to a common overlapping region (at least 400 bp in length), and a phylogenetic tree was built using the Tamura‐nei model and the neighbor‐joining clustering method (with 1000 replicates, and a 50% support threshold) within the Geneious Prime Tree Builder (Geneious Prime). A *Lygus hesperus* COI sequence (acc. NC_024641.1, NCBI) was used as an outgroup. Samples were assigned a taxon according to the presence of the closest reference sequences within the same clade, thus validating or rejecting the identification obtained previously via gross morphology or RAD‐seq alignment rate to a reference genome. The phylogenetic tree is provided in Figure S1.

### 2.5 *trnF* Sequence Analysis

#### 2.5.1 Sampling of Plant DNA

Gut contents were profiled by PacBio sequencing based on the protocol published by (Cooper et al. 2019). Briefly, all DNA samples were subject to amplification of the chloroplast gene *trnF* using primers B49873‐e (GGT TCA AGT CCC TCT ATC CC) and A50272‐F (ATT TGA ACT GGT GAC ACG AG) (Taberlet et al. 1991). Different combinations of barcoded primers (forward and reverse) were used to allow multiplexing samples for sequencing, following the protocol established by Cooper et al. (2019). In addition, ten negative control samples were included to account for any contamination in the reagents. All samples were sent for PacBio sequencing at the Washington State University Laboratory for Biotechnology and Bioanalysis (WA, USA).

#### 2.5.2 Reference Database

We built a custom database to define parameters to identify and classify our sequencing data. The entire RefSeq Plastid genomic database (NCBI, release 219) was downloaded, unzipped and concatenated into a single file. For the next steps we used Mothur (version 1.47; Schloss et al. 2009). We first performed an *in silico* PCR to amplify only the region targeted by our *trnF* primers B49873‐e and A502720‐F. Previously, we had found that most of our PacBio sequences were 400‐600 bp long (Cooper et al. 2019), but there were cases of sequences smaller than 100 bp and greater than 1500 bp. In fact, it is known that the gene *trnF* can greatly vary in size because of repeats and pseudogenes (e.g., Vijverberg and Bachmann 1999; Yan et al. 2019). Based on this, we decided to take a conservative approach for quality‐filtering our data by excluding from our plastid database any sequences smaller than 100 bp, or longer than 10 K bp, which were likely nontarget amplifications or plastids from unicellular organisms (e.g., algae). In addition, we also excluded all sequences with > 5 ambiguities. The median size of sequences passing these filters was 399 bp, ranging from 101 bp to 9119 bp, for a total of 6988 sequences. Next, we used custom scripts to add and format taxonomical rank information to the header section of each sequence, so that it can be read by the DADA2 classifier.

#### 2.5.3 PacBio Data

PacBio data were quality‐trimmed, assembled into ASV and classified using DADA2 v. 1.22.0 (Callahan et al. 2016). Briefly, each sequencing batch (three total) started being processed separately as following: primer sequences were trimmed, and amplicons were removed if they presented unidentified nucleotides (“N”), and/or a quality score smaller than 3, and/or length smaller than 100 bp. In addition, we set TruncQ to 2, causing reads to be truncated at the first nucleotide with a Phred quality score ≤ 2, thereby removing extremely low‐quality sequence tails. We also excluded sequences with a value > 8 for maxEE, i.e., the maximum number of expected errors (EE), with EE defined as sum(10^(−Q/10)^), where Q is the quality score. Next, sequences were dereplicated and we denoised sequences using DADA2 error estimation algorithm, with band size set to 32 bp and the DADA2's error model for PacBio data. All three batches of quality‐trimmed, denoised PacBio sequences were merged, and chimeric sequences were identified and removed using the “removeBimeraDenovo” function in DADA2. Finally, all sequences were classified using our custom database as reference (trnF_db11Feb22.fa), and the DADA2's Bayesian naïve classifier algorithm.

After chimera removal and taxonomical assignment, data were transformed into a phyloseq object (McMurdie and Holmes 2013) and decontaminated using the R package decontam (Davis et al. 2018). We found that a few likely contaminants were present in the control samples, such as sequences identified as *Citrus* and *Pinus*. After further investigation, we found that the water used to dilute the primer solutions was contaminated by these taxa, in addition to other plant and algae taxa. Although these are obvious contaminants, we cannot rule out the possibility that some of these contaminants may also be present as diet items, in particular taxa also found in sampling sites (e.g., *Carya* and *Pinus*). Therefore, we set up our filter to a moderately‐high threshold of sensitivity (0.5) and removed all 15 ASV identified as contaminants from our samples. To further improve taxonomy, we also used the NCBI Blast tool against their nt database to classify the remaining ASVs without a genus ID (e‐value = 1e−30, percent ID 90%–100% for genus identification). Next, we removed any ASV with a classification other than Viridiplantae (Kingdom), or ASVs identified as mosses (Class Bryopsida). Therefore, only spermatophytes were left for downstream analysis.

#### 2.5.4 Diet Diversity Analysis

For diversity analysis, we aggregated ASVs at the genus level. Alpha diversity (Shannon index) was calculated for each species and month of sampling. For beta‐diversity, we accounted for different sampling depth by rarefying samples to the 50% quantile of sample size range, which gave us the best trade‐off between sequencing depth (108 sequences per sample) and sample retention (*n* = 69 samples), using the R package metagMisc (Mikryukov 2026) and 1000 iterations. Permutational multivariate analysis of variance (PERMANOVA) was used to test differences in diet due to sampling location, species identity and/or time of sampling. Data dispersion was tested a priori using *betadisper* (in the vegan R package), and Weighted‐Unifrac distances (calculated by GUnifgrac; Chen et al. 2025) were found to meet homogeneity of variance (*p* > 0.05) for all PERMANOVA tests. Principal Coordinate Analysis (PCoA) was used to evaluate diet dissimilarity among samples, using Weighted‐Unifrac distances (Chen et al. 2025). The function *envfit* from the R package vegan (Oksanen et al. 2026) was used to find plant genera that significantly (*p* < 0.05) correlated with sample ordination space. All these analyses and figures were generated using R packages previously mentioned and the following: Biostrings (Pagès et al. 2025), cowplot (Wilke 2025), DECIPHER (Wright 2016), ggplot2 (Wickham 2016), ggpubr (Kassambara 2026), ggrepel (Slowikowski 2026), ggside (Landis 2025), ggtext (Wilke and Wiernik 2022), gridExtra (Auguie 2017), microbiome (Lahti and Shetty 2012), phangorn (Schliep 2011), RcolorBrewer (Neuwirth 2022), and tidyverse (Wickham et al. 2019).

## 3 Results

### 3.1 Sample Identification

A total of 118 individuals were identified via molecular analysis: 60 individuals collected in March and May–October 2020 and 58 individuals in July–October 2021 (Table S1). Molecular data (RAD‐seq) revealed that 67 of these samples were *Dallasiellus lugubris* , 34 were *Pangaeus bilineatus* , and the species identification of 17 samples could not be determined either by alignment to our reference genomes nor by BLAST against the NCBI nt/nr database (Table S1). Our *COI* sequence analysis further validated these findings using a subset of the samples (Figure S1). We obtained both diet data and RAD‐seq data for all of these samples. An additional 32 field‐collected samples and 10 captive individuals were identified as *Pangaeus bilineatus* via visual diagnostic, but not sequenced for RAD‐seq. Most *P. bilineatus* individuals were collected in the summer (peaking in June 2020), whereas most *D. lugubris* were collected in the fall (peaking in September 2021) (Figure S2).

### 3.2 Diet Analysis

We obtained 120,041 raw reads for 160 individuals, 115,389 for 150 field collected samples and 4652 reads for 10 *P. bilineatus* individuals reared in captivity. After quality‐trimming, denoising and removing contaminants, all captive *P. bilineatus* samples were kept, with a total of 3944 reads, whereas 138 samples were kept for field collected individuals, with a total of 90,959 reads. Among field samples kept in the dataset, 61 were identified as *D. lugubris* , 62 as *P. bilineatus* , and 15 were classified as “unknown.” Using our custom *trnF* database and the DADA2 Bayesian classifier, we identified 180 ASVs to Family and 158 to Genus. After manually curating results and adding results obtained via NCBI's BLAST (nt/nr database), we were able to identify 186 ASVs to the Genus level, and 188 at the Family level. *Pangaeus bilineatus* and *D. lugubris* samples were found to contain between 43 and 50 plant genera, respectively, with broad variation in diet diversity among sample sites and years (Figure S2; Tables S2 and S3).

**FIGURE 1:**
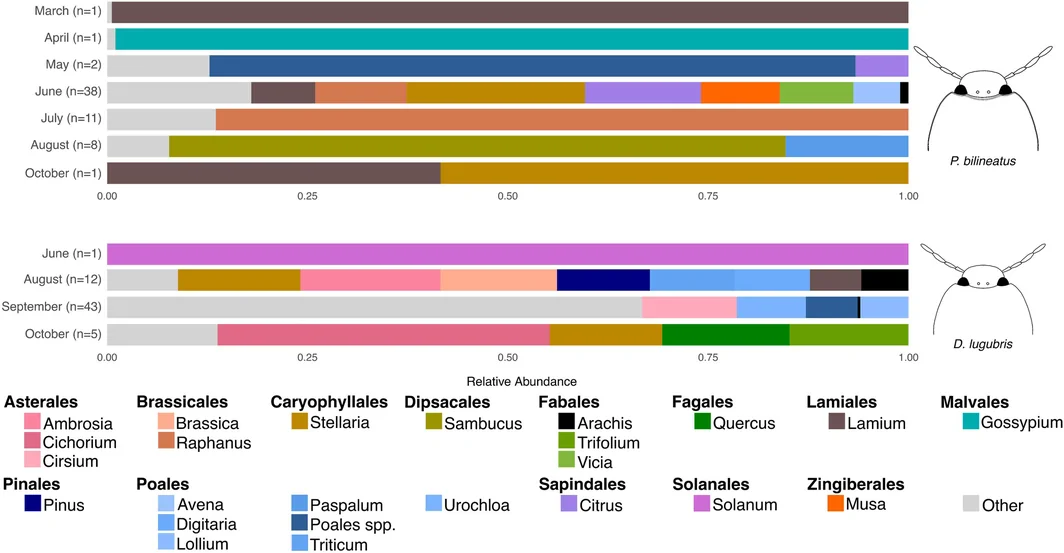
Relative abundance of the average number of times plant genera were detected in *P. bilineatus* and *D. lugubris* . Every plant genus with a relative (average) abundance smaller than 5% was grouped as “Other,” with exception of *Arachis* that is always represented in black.

**FIGURE 2:**
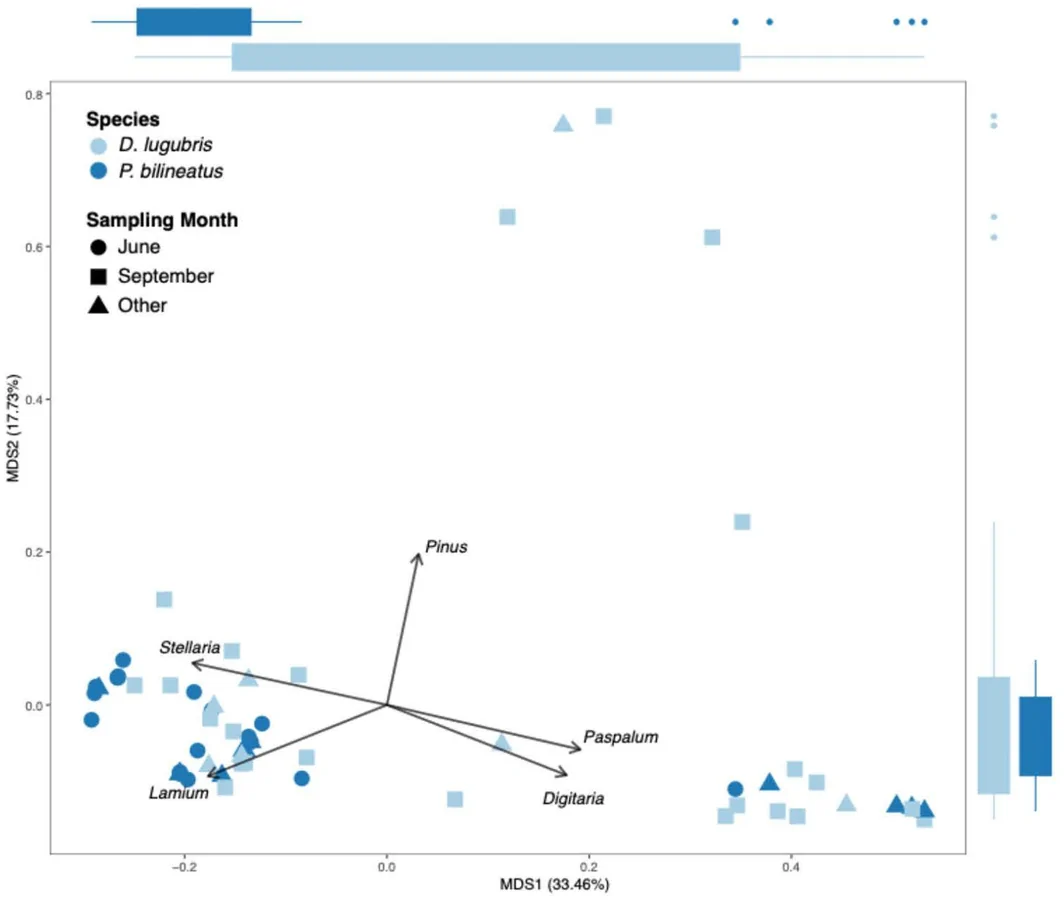
Dissimilarity of plant composition detected in samples of *P. bilineatus* and *D. lugubris* (Principal Coordinates Analysis, samples rarefied to the median sample size, using Unifrac distances at alpha = 0.5). Most *D. lugubris* were sampled in September, whereas *P. bilineatus* individuals are mostly represented by samples collected in June, which may be correlated with seasonal differences in plant phenology. The *envfit* function from the vegan package is used here to find plant taxa that are mostly correlated with the ordination space (*p* < 0.05), and the length of arrows corresponds to the scale of correlation (longer, higher correlation than shorter, weaker correlations).

**FIGURE 3:**
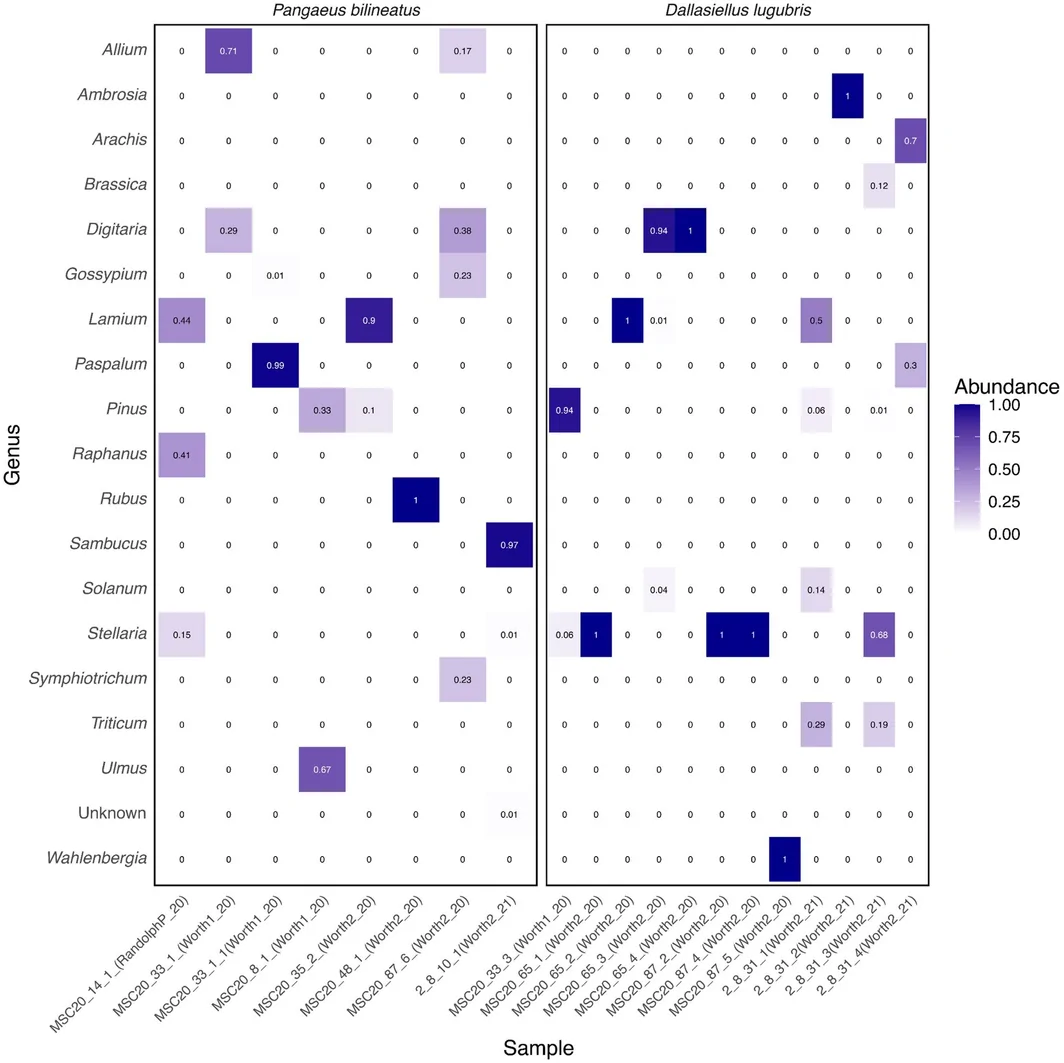
Heatmap depicting the diet composition of *P. bilineatus* and *D. lugubris* for the month of August. “Abundance” is the proportion of *P. bilineatus* or *D. lugubris* samples found to contain plant DNA.

No significant difference in diet composition was found to associate with burrower bug species (permutations for species diet performed within sampling site and blocked by year of sampling; (*F*_5,60_ = 1.099, *R* ^2^ = 0.085, *p* = 0.330)), but differences were marginally associated with sampling month (*F*_7,60_ = 1.415, *R* ^2^ = 0.154, *p* = 0.045) (Figures 1 and 2). This may be explained by the fact that the abundance of each burrower bug species peaked at a different time of the year (Figure S2), coinciding with changes in the plant community phenology in this region (Pearson 2019). Wild weeds and herbaceous plants, such as *Stellaria* (e.g., chickweeds), *Raphanus* (e.g., radishes), *Paspalum* (e.g., crowngrass), and *Lamium* (e.g., dead‐nettles), as well as a few tree species, such as *Carya* (e.g., pecans, hickory) and *Pinus* (e.g., pine, conifers), were among the most common plants detected in both *P. bilineatus* and *D. lugubris* (Table S2). Genera associated with ornamental or commercial crops were also among those detected in high abundance, such as *Musa* (e.g., ornamental, hardy banana, Figure S4), *Cichorium* (e.g., chicory), and *Sesamum* (e.g., sesame, Figure S5). Peanuts (*Arachis*) was seldom detected, being recorded only four times in *D. lugubris* (reads per sample, *n* = 8, *n* = 17, *n* = 10, and *n* = 30) and twice (*n* = 35 reads and *n* = 2 reads) in *P. bilineatus* . As a contrast, peanut DNA was detected in all 10 samples from captive *P. bilineatus* , being the sole item detected in 4 samples, and over 99.78% of all items detected in the other six samples (see Table S2). To try to disentangle diet differences from suitable hosts availability, we also compared diet composition obtained only for August, when these species were sampled in comparable numbers, both years (*n* = 7 *Pangaeus* and 8 *Dallasiellus*, in 2020, and *n* = 1 *Pangaeus* and 4 *Dallasiellus*, in 2021), and also found no significant difference in their diets (*F*_1,19_ = 1.110, *R* ^2^ = 0.06, *p* = 0.335 (Bray–Curtis), *F*_1,19_ = 1.204, *R* ^2^ = 0.06, *p* = 0.222 (Jaccard)), homogeneity of variance met in both analyses (*p* > 0.05), with year of sampling not significant in both cases (*F*_1,19_ = 1.01, *R* ^2^ = 0.05, *p* = 0.432 (Bray–Curtis), and *F*_1,19_ = 1.04, *R* ^2^ = 0.05, *p* = 0.392 (Jaccard)) (Figure 3). Nevertheless, there was no single plant that was detected consistently across all individuals, either for *Pangaeus* or *Dallasiellus*, with *Stellaria* being the plant with highest incidence across individuals of the same species, detected in 5 out of 12 *D. lugubris* individuals (Figure 3).

### 3.3 Population Structure Analysis

**FIGURE 4:**
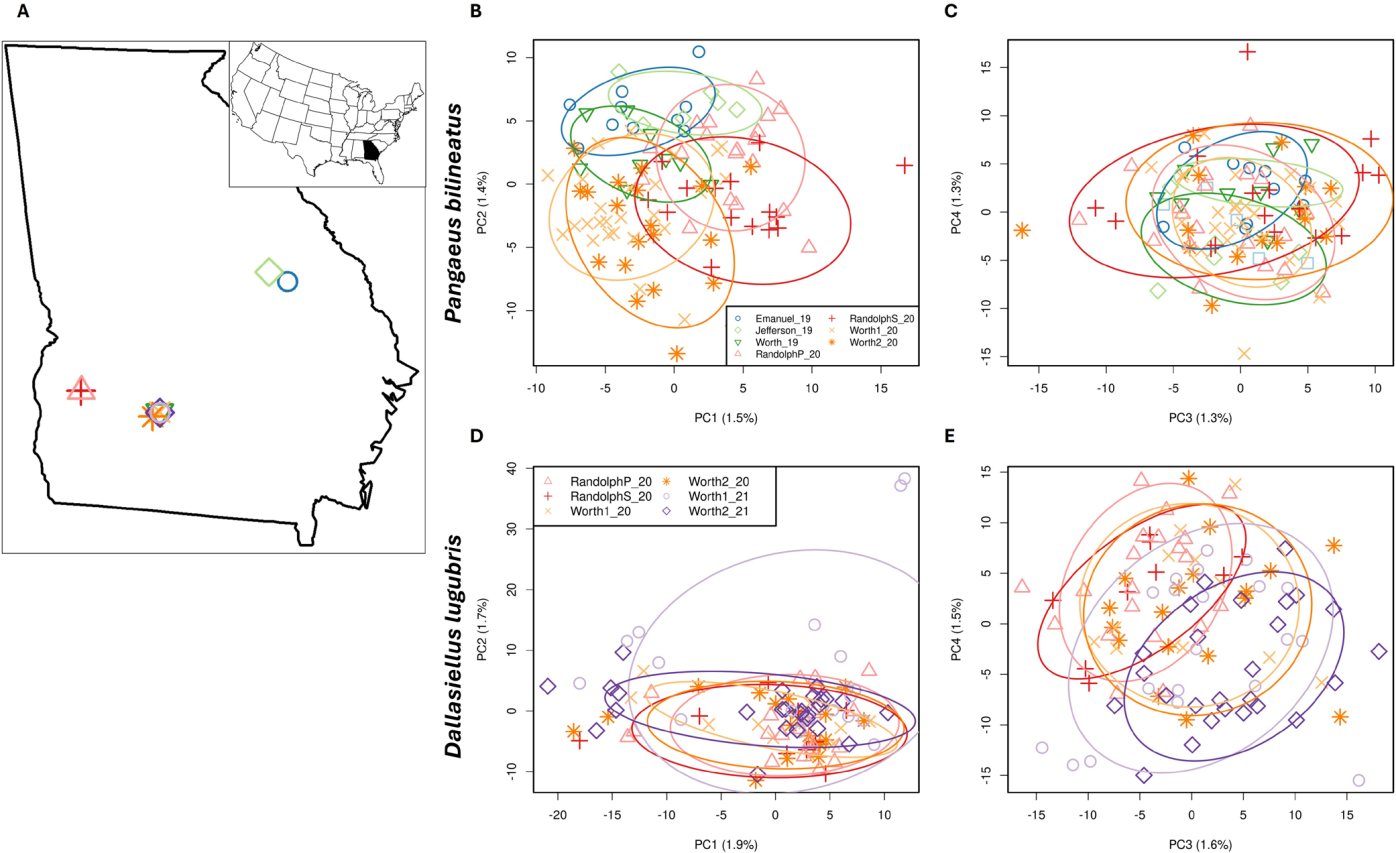
Map of sampling locations (A) and Principal Components Analysis biplots made from SNP genotypes for *Pangaeus bilineatus* (B, C) and *Dallasiellus lugubris* (D, E). Samples are denoted by site (county) and year of collection.

**FIGURE 5:**
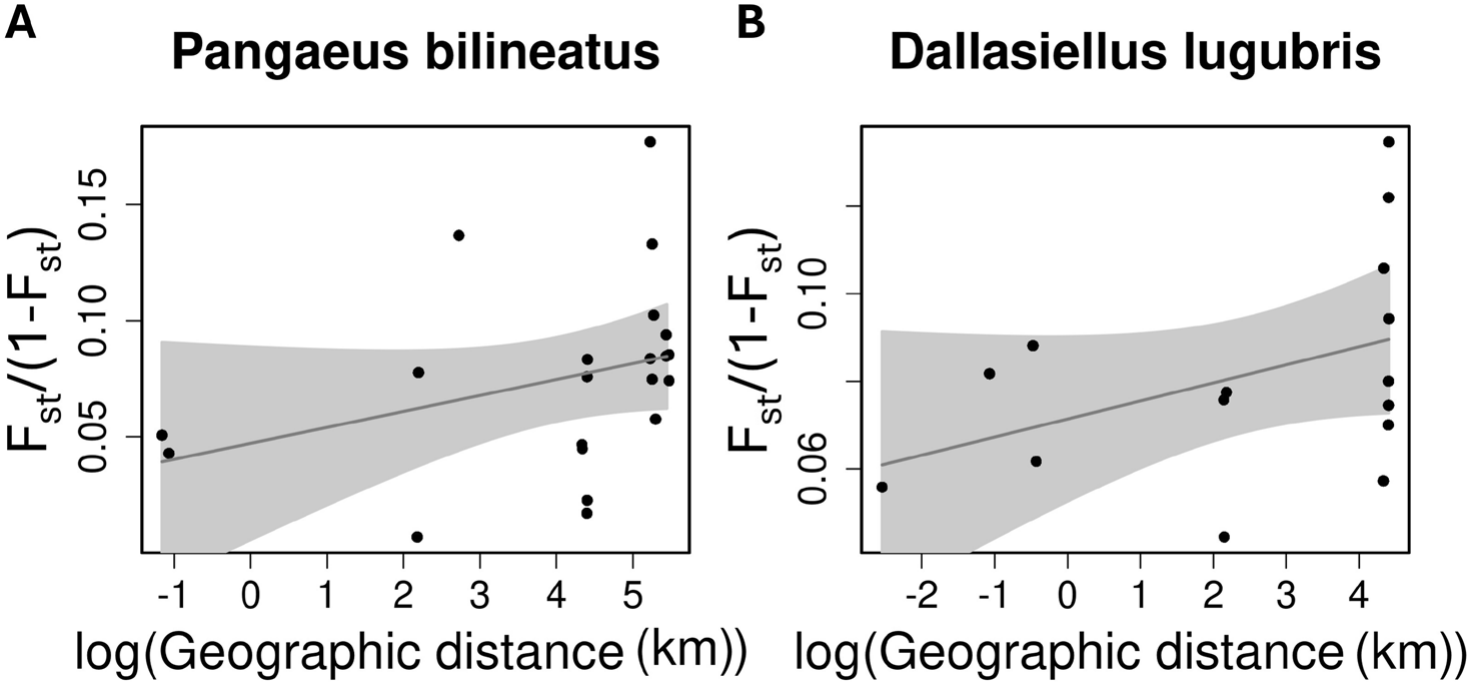
Relationship between genetic distance and geographic distance for *Pangaeus bilineatus* (A) and *Dallasiellus lugubris* (B). Gray bands depict 95% confidence intervals.

Principal Components Analysis revealed a small degree of separation of *P. bilineatus* samples by location along PC1 (explaining 1.5% of variation) and PC2 (explaining 1.4% of variation), with no further separation along PCs 3 and 4 (Figure 4A,B). In contrast, *D. lugubris* samples showed no separation by location along PC1 (explaining 1.9% of variation) or PC2 (explaining 1.7% of variation) (Figure 4C). However, some separation by year was evident along PC3 (explaining 1.6% of variation) and PC4 (explaining 1.5% of variation) (Figure 4D). For *P. bilineatus* and *D. lugubris* , sPCA revealed significant global spatial genetic structure ( *P. bilineatus* Monte Carlo permutation test: standardized statistic = 5.81, *p* = 0.005; *D. lugubris* Monte Carlo permutation test: standardized statistic = 5.21, *p* = 0.005) but no significant local spatial genetic structure ( *P. bilineatus* standardized statistic = −1.28, *p* = 0.90; *D. lugubris* standardized statistic = −2.15, *p* = 0.975), indicating weak but spatially organized genetic differentiation across the study region (Figure S6). Pairwise *F*_ST_ values ranged from 0.017 to 0.15 for *P. bilineatus* (Table S4), and from 0.043 to 0.119 for *D. lugubris* (Table S5). Mantel tests provided evidence consistent with weak isolation by distance for both species ( *P. bilineatus* : Mantel *r* = 0.33, *p* = 0.034; *D. lugubris* : Mantel *r* = 0.41, *p* = 0.038) (Figure 5).

## 4 Discussion

New insect threats to agriculture are constantly emerging, creating the need for knowledge about their host breadth, dispersal, and other factors contributing to their pest status. Our analysis of an emergent pest of peanut through molecular gut content analysis revealed just 3 out of 62 *P. bilineatus* were positive for peanut (*Arachis*). Surprisingly, *D. lugubris* , another burrower bug species found to be similarly abundant (though at different times of year), was found to feed on peanut, though still seldom for this species as well (4 out of 61). While peanut‐feeding was rare among adult bugs sampled, the ability of this species to feed on alternative hosts may help explain their permanence in the region throughout the year. Though co‐occurrence in peanut fields of several burrowing bug species is not uncommon (Smith and Pitts 1974; Chapin and Thomas 2003), economic losses have overwhelmingly been attributed to *P. bilineatus* (Aigner et al. 2021). Thus, the abundance of *D. lugubris* at our sample sites in southern Georgia late in the season combined with evidence of peanut‐feeding suggests that economic losses in peanut crops from burrower bugs could be attributed to a species complex, rather than to *P. bilineatus* alone. Furthermore, our analysis suggests that investigation of the diets of all burrower bug species occurring in peanut fields, particularly during peanut pod maturation, is needed to determine the extent of damage caused by these other species.

Characterizing the host plant breadth of emergent agricultural pests is important for understanding where and when they occur in farmscapes. For example, identifying wild hosts of the boll weevil ( *Anthonomus grandis* ) and removing them from habitats surrounding cotton fields is crucial to continued efforts to manage boll weevil in the southern US (Sánchez‐Reyes et al. 2022). Our molecular gut content analysis identified 188 plant families among DNA extracted from burrower bug specimens. These families included wild forbs, grasses, and trees, as well as ornamentals and an impressive variety of agricultural crops (from 13 genera). That *P. bilineatus* feeds on a variety of crops is not wholly unexpected. *Pangaeus bilineatus* has been documented damaging cotton seedlings, peppers, strawberry, spinach, and wheat seedlings, in addition to peanut (Schwertner and Nardi 2015; Aigner et al. 2021). Our analysis recapitulated these findings except for strawberry (which is uncommon near our study sites). However, the sheer breadth of plant taxa emerging from our study, for both *P. bilineatus* and *D. lugubris* , for which little about host breadth is known, demonstrates the great value of molecular approaches to characterizing the host plant breadth of emergent agricultural pests, even if subsequent laboratory studies are needed to confirm the importance of these plants throughout the insects' life cycle. The broad diet generalism exhibited by these two species throughout the growing season may be a key factor contributing to the sporadic nature of economic losses in peanuts attributable to burrower bugs.

In cases where sufficient genetic variation exists to examine population structure, tools like RAD‐seq can provide useful insights into population‐level dispersal throughout farmscapes. For example, RAD‐seq data revealed geographic barriers separating lineages of the boll weevil (Raszick et al. 2021), and a similar tool (genotyping‐by‐sequencing) identified host plant connectivity as a key determinant of genetic differentiation and diversity among Colorado potato beetle ( *Leptinotarsa decemlineata* ) populations (Crossley et al. 2019). Our RAD‐seq analysis revealed evidence consistent with weak isolation‐by‐distance, or a stepwise pattern of population dispersal among burrower bug populations in southern Georgia, wherein populations separated by greater geographic distance generally exhibit greater genetic differentiation (Slatkin 1993). Slightly greater genetic differentiation was evident from PCA. sPCA, and *F*_ST_ among *P. bilineatus* populations, suggesting that *D. lugubris* may be relatively more widely dispersive among farmscapes. The dispersal behavior of burrower bugs has not been studied, with species like *P. bilineatus* instead being known for their tendency to crawl and seek shelter during daylight near the soil surface and at the bases of plants (Schwertner and Nardi 2015). Burrower bugs clearly fly to light attractants at night, so presumably disperse more widely by flight at night whether fleeing predators or seeking mates, oviposition sites, or new host plants.

The nature of our study system and of our data collection preclude firm conclusions about some aspects of our study. For instance, we found some separation in host plant composition between *P. bilineatus* and *D. lugubris* that seems to be due to seasonal plant phenology rather than dietary preferences. However, the large variation in diet composition even with these species makes it uncertain whether we were able to detect true diet preferences as opposed to occasional feeding by dispersing adult individuals. We also detected *Musa* sp. (e.g., banana, ornamental) in 7 individuals (including three individuals from our captive colony) and *Sesamum* sp. (e.g., sesame, in 11 individuals) (Figures S3 and S4). Despite efforts to remove potential contaminants bioinformatically, we cannot exclude the possibility that these taxa reflect the environment in which samples were transported and processed. Still, banana (ornamental and edible) and sesame cultivation are not wholly unknown to our study region, in some instances even being promoted as novel, alternative crops in southern Georgia (Ray 2013; Passmore 2018; Banana Tree Nursery 2026). Ornamental bananas were also observed at dwellings near our light traps (MSC personal observation). In addition, because we sequenced a chloroplast gene, *trnF*, our analysis should not be sensitive to contamination by pollen, which typically does not carry chloroplast DNA (Mogensen 1996). However, pines and grasses can carry chloroplast DNA in their pollen (Mogensen 1996; Petit et al. 2005), so confirmation of these taxa in burrower bug diets requires experimental evaluation. Lastly, our samples comprised adults that flew into light traps, thus precluding assessment of nymphal diets. If nymphs are the primary damaging stage of peanuts during pod maturation, this may explain the apparent lack of peanut‐feeding among sampled insects, despite their status as a significant peanut pest.

Amidst ever‐changing weather patterns and continually evolving agricultural management practices, new agricultural insect pest threats will continue to emerge and require fast and effective responses by growers and pest management practitioners (Meehan et al. 2011; Deutsch et al. 2018; Ma et al. 2021). Where the diet breadth of emergent pests is unknown, molecular gut content analysis can provide helpful snapshots of diet composition across sites and through time (Fluch et al. 2024). Where the appropriate scales over which to consider pest populations are uncertain, population genomics approaches can allow inferences of population connectivity throughout landscapes (Perry et al. 2020). Here, the combination of molecular gut content and RAD‐seq analysis revealed a potential pest complex where previously only one species was in view and suggested an opportunity to uncover landscape factors influencing connectivity among pest populations. Taken together, we suggest that responses to emerging pest challenges can benefit from insights generated by population genomics techniques, opening up new avenues for research and supporting efforts to tailor management strategies for novel pests.

## Author Contributions

**Pedro A. P. Rodrigues:** conceptualization (equal), data curation (equal), formal analysis (equal), methodology (equal), visualization (equal), writing – original draft (equal). **Michael S. Crossley:** conceptualization (equal), data curation (equal), formal analysis (equal), methodology (equal), visualization (equal), writing – original draft (equal). **Benjamin L. Aigner:** data curation (supporting), writing – review and editing (supporting). **William Rodney Cooper:** methodology (supporting), resources (supporting), writing – review and editing (supporting). **David Scott Carlson:** data curation (supporting), writing – review and editing (supporting). **Pamela Sapp:** data curation (supporting), writing – review and editing (supporting). **Mark R. Abney:** data curation (supporting), methodology (supporting), resources (supporting), writing – review and editing (supporting). **William E. Snyder:** resources (supporting), supervision (supporting), writing – original draft (supporting), writing – review and editing (supporting).

## Funding

This work was supported by the U.S. Department of Agriculture, DEL00854.

## Conflicts of Interest

The authors declare no conflicts of interest.

## Supporting information


**Figure S1:** Sample molecular identification via COI and phylogeny. Sample sequences (green) are compared to reference sequences downloaded from NCBI (blue) of related burrower bug species, as well as reference sequences we generated (red) from specimens identified by a specialist (Mark Abney, University of Georgia). A sample that failed to be identified by RAD‐seq data, sample 19,301, is also included. Samples identified as *Pangaeus bilineatus* are highlighted by a yellow background, *Dallasiellus lugubris* by a green background. Phylogenetic tree built using the neighbor‐joining clustering method and the Tamura‐Nei nucleotide substitution model.
**Figure S2:** Number of *Pangaeus bilineatus* and *Dallasiellus lugubris* collected in light traps in southern Georgia, aggregated by time of year (Spring samples included March 20–June 16; Summer samples ranged from June 22–September 7; and Fall samples were collected between September 22–October 27).
**Figure S3:** Violin plots depicting the diet diversity (Shannon Index) of (a) *P. bilineatus* and (b) *D. lugubris* among sites aggregated across sample dates. Thick lines depict medians, box edges depict interquartile ranges, whiskers depict ranges minus outliers, dots depict outliers, and violin outlines depict the smoothed probability density function among bug samples.
**Figure S4:** Abundance of ASVs aligning to *Musa* among *P. bilineatus* samples.
**Figure S5:** Abundance of ASVs aligning to *Sesamum* among *P. bilineatus* samples.
**Figure S6:** Spatial principal component analysis (sPCA) of *P. bilineatus* (A–C) and *D. lugubris* (D–F) RADseq data. (A, D) Eigenvalue spectrum showing positive (global) and negative (local) spatial structure. (B, E) Spatial distribution of scores on the first positive sPCA axis (sPC1). Symbol size is proportional to the absolute value of the score and shading indicates score sign and magnitude. Coordinates were jittered slightly for visualization because multiple individuals were sampled from identical locations. (C, F) Distribution of sPC1 scores by population. Positive and negative scores indicate opposing ends of the primary spatial genetic gradient. Together, these results suggest weak‐to‐moderate but geographically coherent population genetic structure across the sampled range.
**Table S1:** Sample metadata information, including negative controls, location site, date and identification whether via molecular methods (percent alignment to draft genomes) or visual diagnostic.
**Table S2:** Summary of plant taxa detected in *Pangaeus bilineatus* and *Dallasiellus lugubris* , with OTUs collapsed at the Genus‐level.
**Table S3:** Pacbio sequencing data summarized as OTU detected in each sample with corresponding taxonomical classification and metadata information.
**Table S4:** Pairwise Weir and Cockerham *F*_ST_ values for *Pangaeus bilineatus* samples. Population labels indicate county and year of collection (e.g., Worth1_20 came from Worth County in 2020).
**Table S5:** Pairwise Weir and Cockerham *F*_ST_ values for *Dallasiellus lugubris* samples. Population labels indicate county and year of collection (e.g., Worth1_20 came from Worth County in 2020).

## Data Availability

Molecular gut content analysis data are available in the Supporting Information, and code is available on GitHub (https://github.com/PedroDaPos/Burrower‐Bugs‐diet‐and‐pop‐gen). RAD‐seq reads are available on NCBI SRA (PRJNA1481662); PacBio sequencing reads for gut content analysis are available on NCBI SRA (SAMN62116268 through SAMN62116444), and COI sequences and draft genomes ( *P. bilineatus* and *D. lugubris* ) are available on FigShare (10.6084/m9.figshare.33145238).
