# Supplementary material for: Population Genomics Approaches Identify a Cryptic, Emerging Generalist Pest Complex
Source: Ecol Evol. 2026 Sep 11;16(9):e74347. doi: 10.1002/ece3.74347 (PMC13564106; doi:10.1002/ece3.74347)
Supplement: Supplementary file 1 [file ECE3-16-e74347-s001.zip › ece374347-sup-0001-Supinfo.docx]

**Supplementary Online Material for**

Population genomics approaches identify a cryptic, emerging generalist pest complex

Pedro A. P. Rodrigues, Michael S Crossley, Benjamin L. Aigner, W Rodney Cooper, D. Scott Carlson, Pamela Sapp, Mark R. Abney, William E. Snyder

**Supplementary Tables**

**Supplementary Table S1.** Sample metadata information, including negative controls, location site, date and identification whether via molecular methods (percent alignment to draft genomes) or visual diagnostic.

**Supplementary Table S2.** Summary of plant taxa detected in *Pangaeus bilineatus* and *Dallasiellus lugubris*, with OTUs collapsed at the Genus-level.

**Supplementary Table S3.** Pacbio sequencing data summarized as OTU detected in each sample with corresponding taxonomical classification and metadata information.

**Supplementary Table S4.** Pairwise Weir and Cockerham F_ST_ values for *Pangaeus bilineatus* samples. Population labels indicate county and year of collection (e.g., Worth1_20 came from Worth County in 2020).

|  | **Worth_19** | **Emanuel_19** | **Jefferson_19** | **Worth1_20** | **RandolphP_20** | **RandolphS_20** |
| --- | --- | --- | --- | --- | --- | --- |
| **Emanuel_19** | 0.117 |  |  |  |  |  |
| **Jefferson_19** | 0.150 | 0.120 |  |  |  |  |
| **Worth1_20** | 0.048 | 0.070 | 0.077 |  |  |  |
| **RandolphP_20** | 0.071 | 0.079 | 0.078 | 0.017 |  |  |
| **RandolphS_20** | 0.077 | 0.069 | 0.086 | 0.022 | 0.041 |  |
| **Worth2_20** | 0.072 | 0.054 | 0.093 | 0.007 | 0.045 | 0.043 |

**Supplementary Table S5.** Pairwise Weir and Cockerham F_ST_ values for *Dallasiellus lugubris* samples. Population labels indicate county and year of collection (e.g., Worth1_20 came from Worth County in 2020).

|  | **Worth1_20** | **RandolphP_20** | **RandolphS_20** | **Worth2_20** | **Worth2_21** |
| --- | --- | --- | --- | --- | --- |
| **RandolphP_20** | 0.065 |  |  |  |  |
| **RandolphS_20** | 0.109 | 0.076 |  |  |  |
| **Worth2_20** | 0.072 | 0.054 | 0.096 |  |  |
| **Worth2_21** | 0.058 | 0.069 | 0.086 | 0.043 |  |
| **Worth1_21** | 0.081 | 0.074 | 0.119 | 0.070 | 0.053 |

**Supplementary Figures**

**Supplementary Figure S1.** Sample molecular identification via COI and phylogeny. Sample sequences (green) are compared to reference sequences downloaded from NCBI (blue) of related burrower bug species, as well as reference sequences we generated (red) from specimens identified by a specialist (Mark Abney, University of Georgia). A sample that failed to be identified by RAD-seq data, sample 19301, is also included. Samples identified as *Pangaeus bilineatus* are highlighted by a yellow background*, Dallasiellus lugubris* by a green background. Phylogenetic tree built using the neighbor-joining clustering method and the Tamura-Nei nucleotide substitution model.

**Supplementary Figure S2.** Number of *Pangaeus bilineatus* and *Dallasiellus lugubris* collected in light traps in southern Georgia, aggregated by time of year (Spring samples included March 20-June 16; Summer samples ranged from June 22-September 7; and Fall samples were collected between September 22-October 27).

**Supplementary Figure S3.** Violin plots depicting the diet diversity (Shannon Index) of (a) *P. bilineatus* and (b) *D. lugubris* among sites aggregated across sample dates. Thick lines depict medians, box edges depict interquartile ranges, whiskers depict ranges minus outliers, dots depict outliers, and violin outlines depict the smoothed probability density function among bug samples.

**Supplementary Figure S4.** Abundance of ASVs aligning to *Musa* among *P. bilineatus* samples

**Supplementary Figure S5.** Abundance of ASVs aligning to *Sesamum* among *P. bilineatus* samples

**Supplementary Figure S6.** Spatial principal component analysis (sPCA) of *P. bilineatus* (A-C) and *D. lugubris* (D-F) RADseq data. (A, D) Eigenvalue spectrum showing positive (global) and negative (local) spatial structure. (B, E) Spatial distribution of scores on the first positive sPCA axis (sPC1). Symbol size is proportional to the absolute value of the score and shading indicates score sign and magnitude. Coordinates were jittered slightly for visualization because multiple individuals were sampled from identical locations. (C, F) Distribution of sPC1 scores by population. Positive and negative scores indicate opposing ends of the primary spatial genetic gradient. Together, these results suggest weak-to-moderate but geographically coherent population genetic structure across the sampled range.
